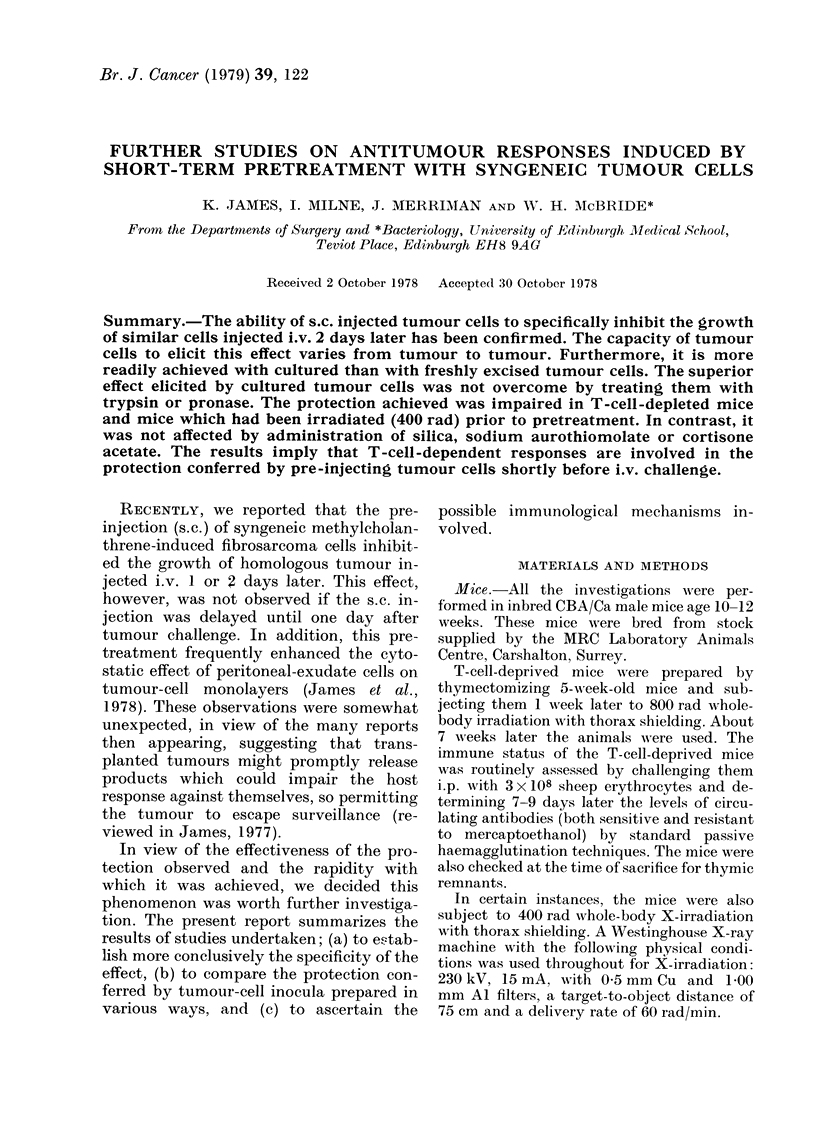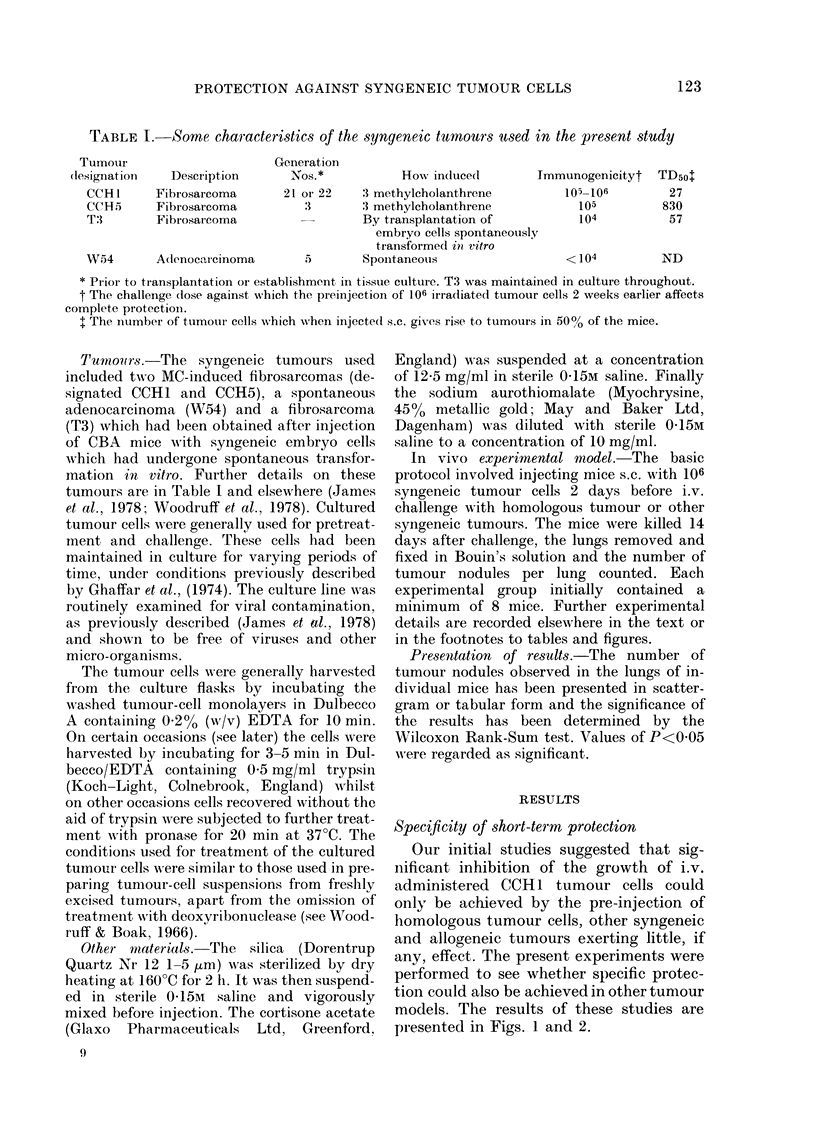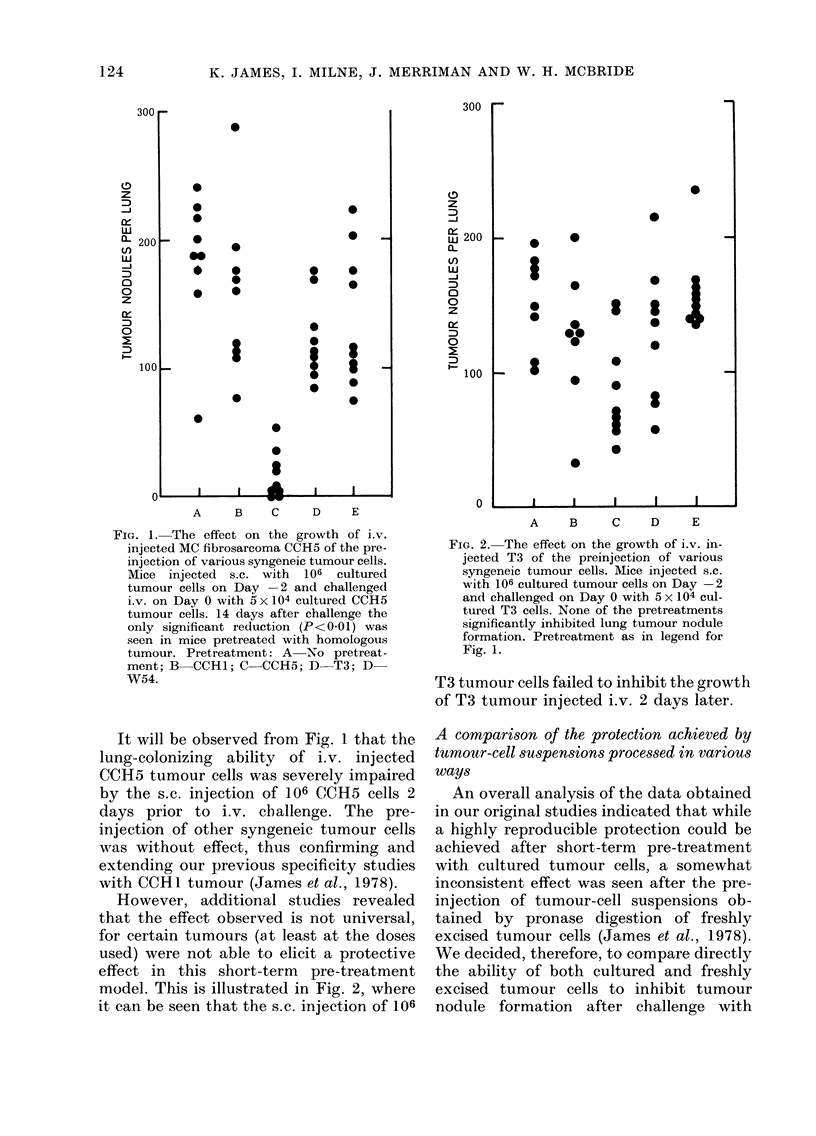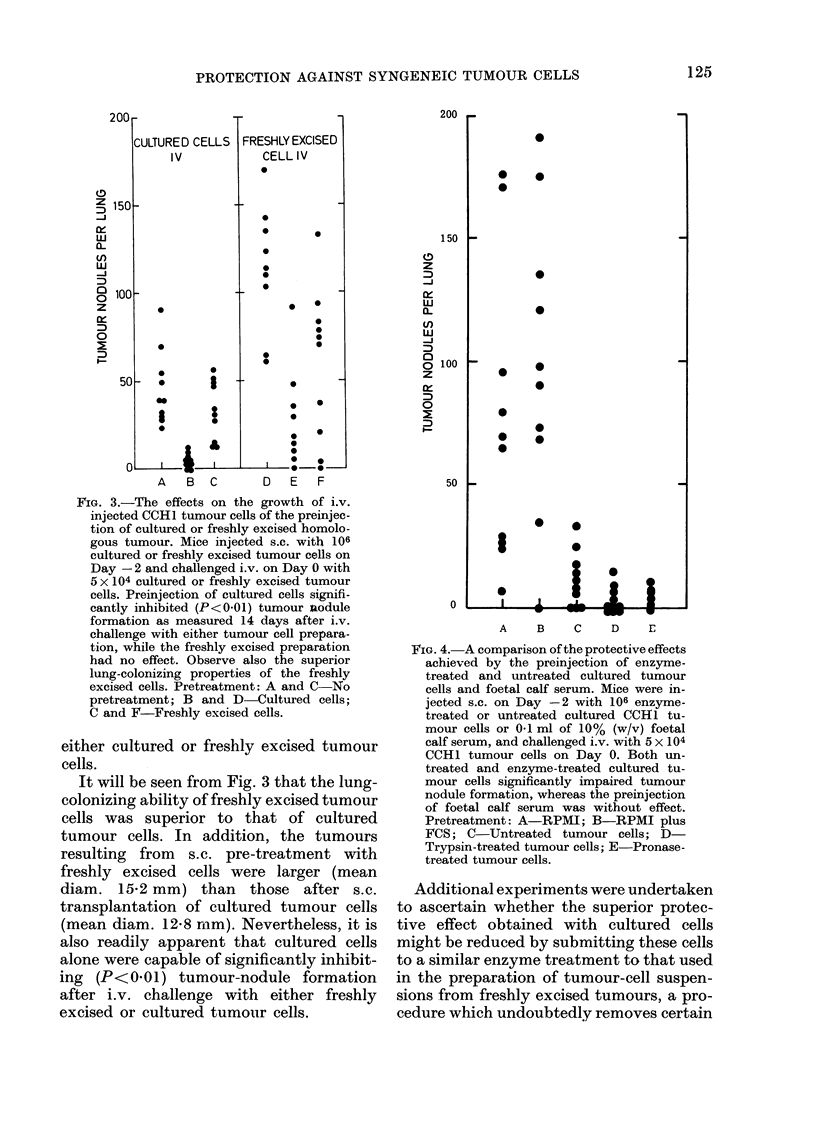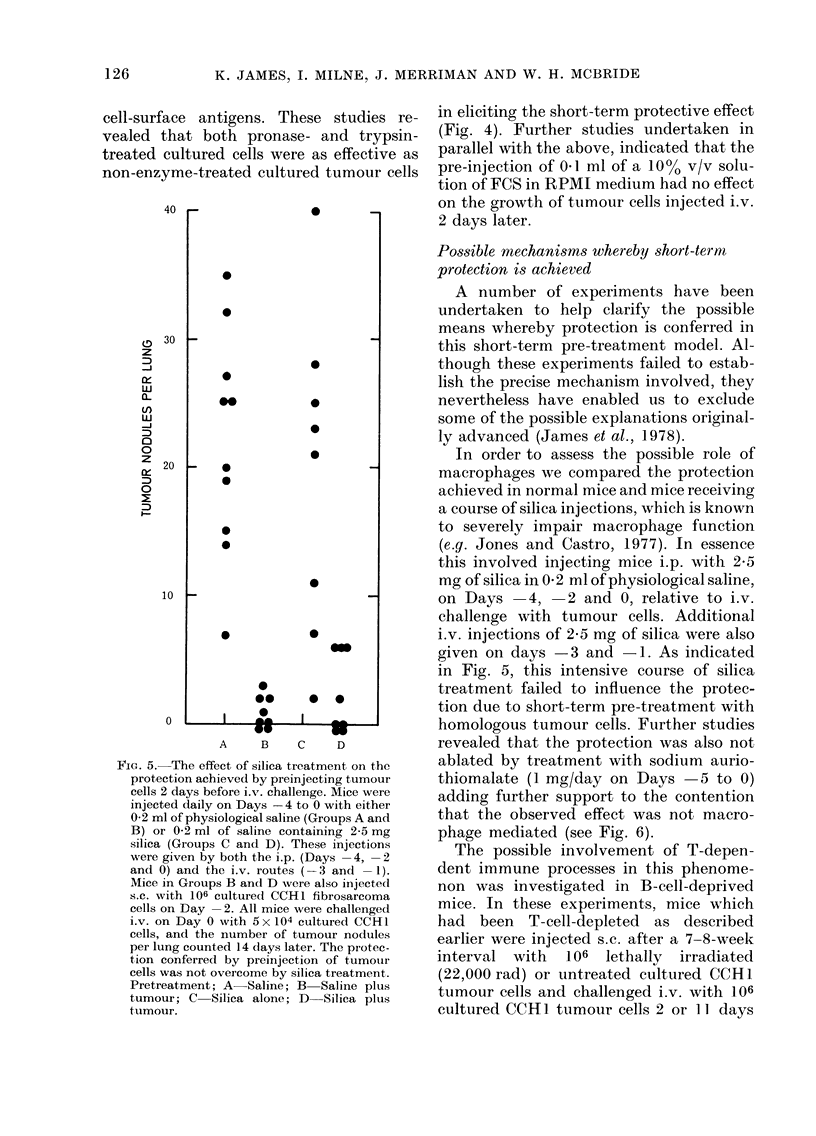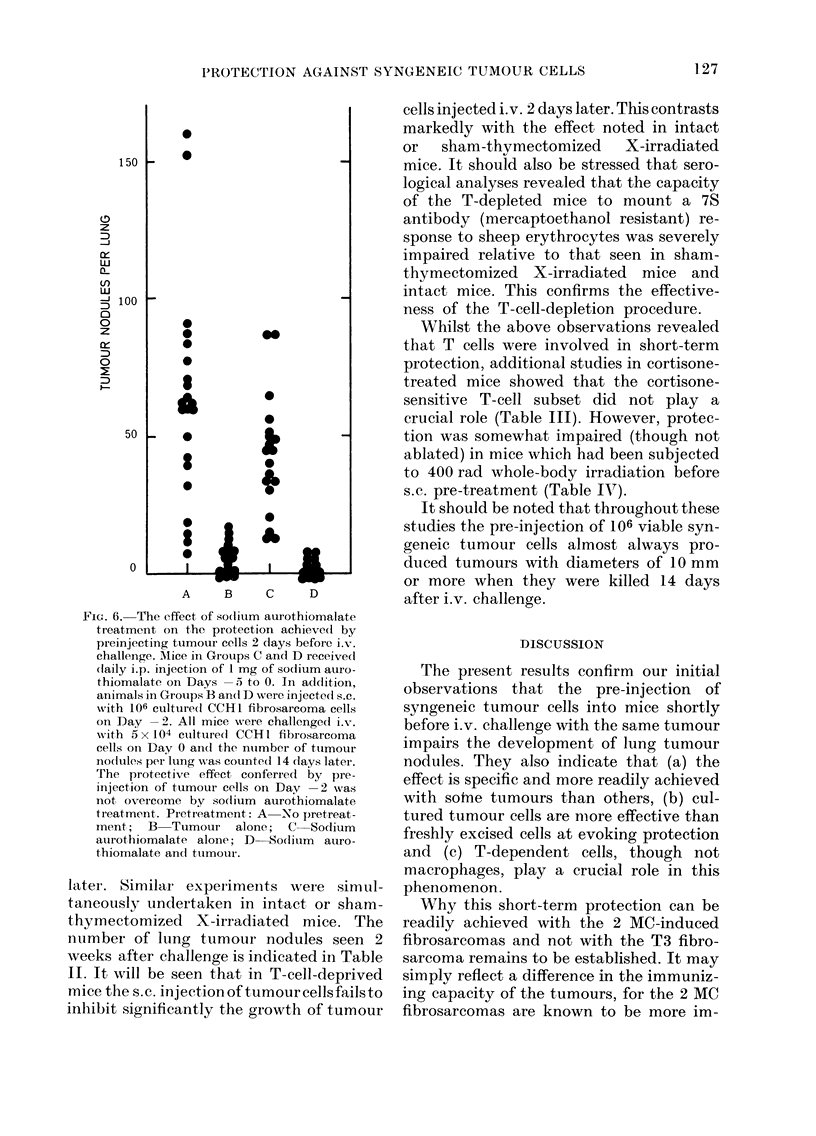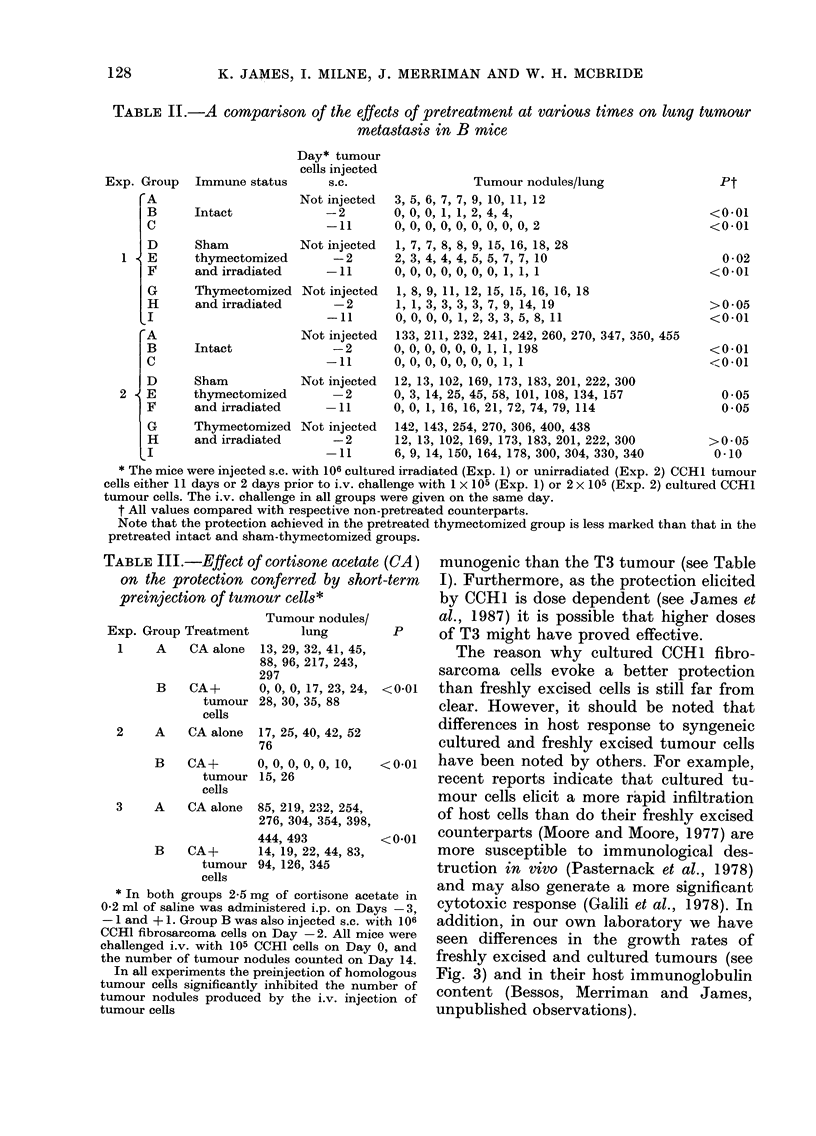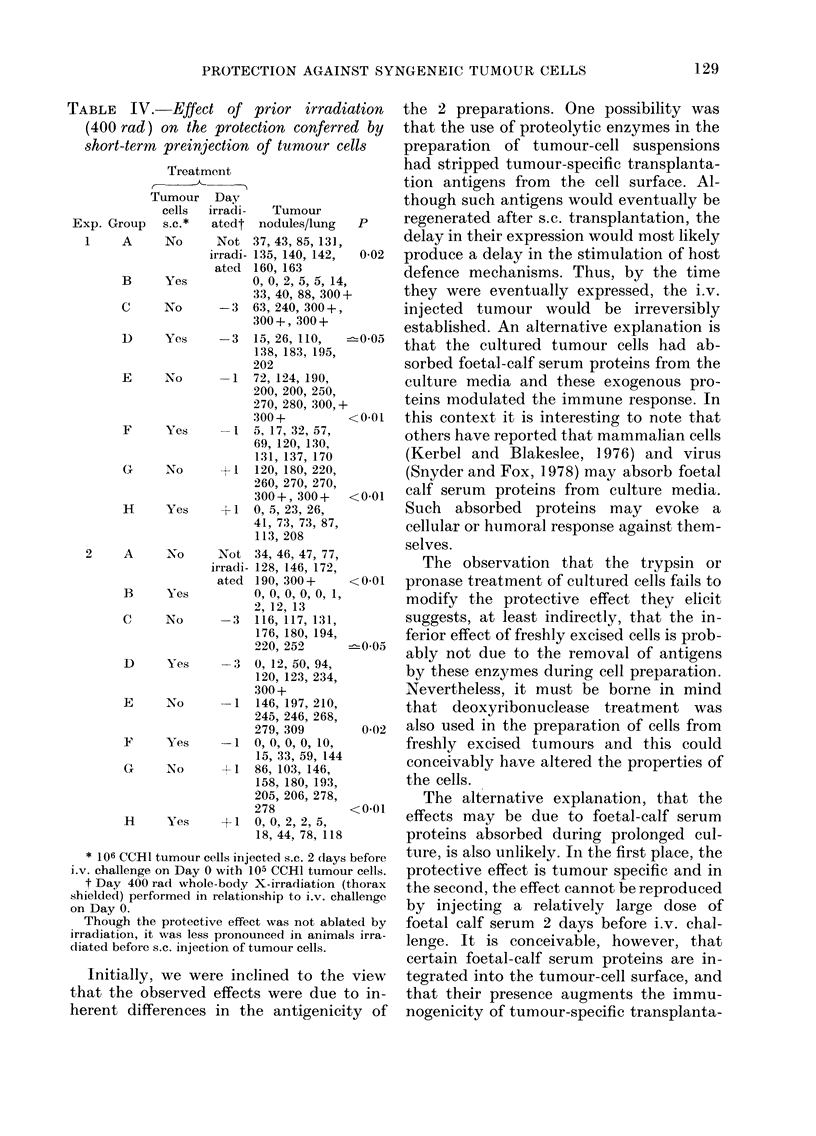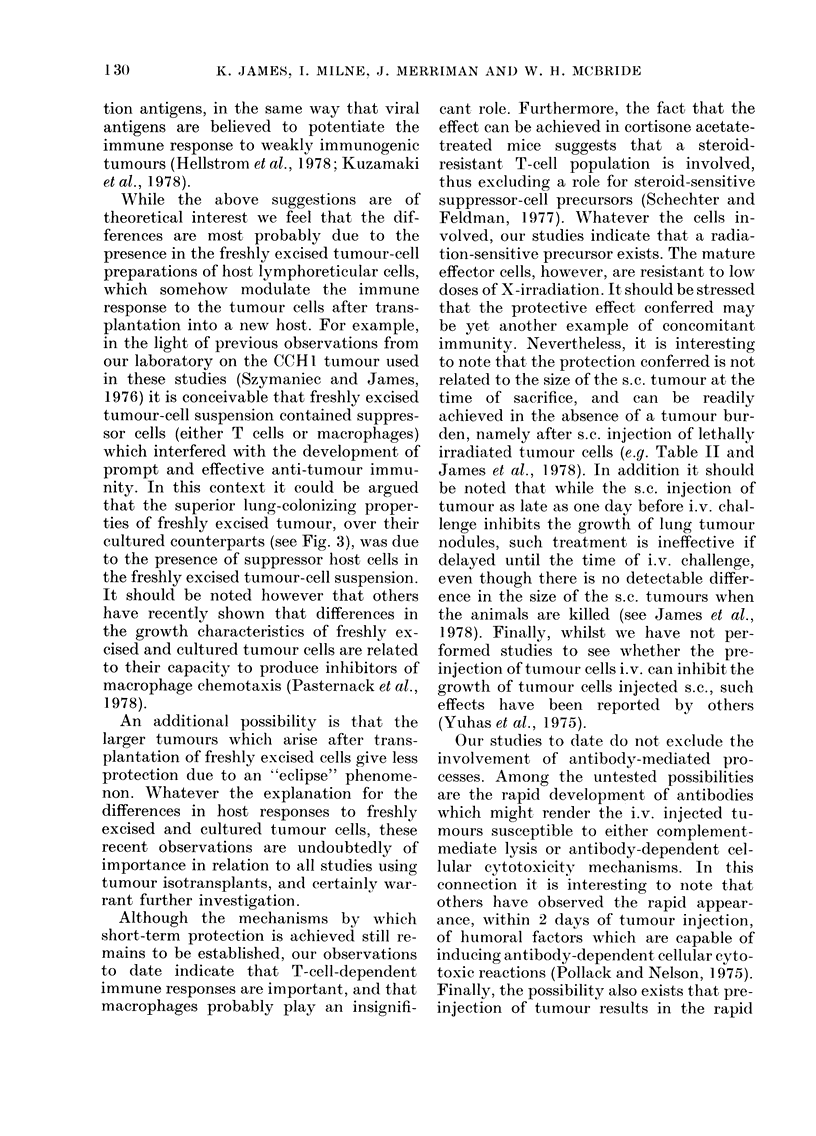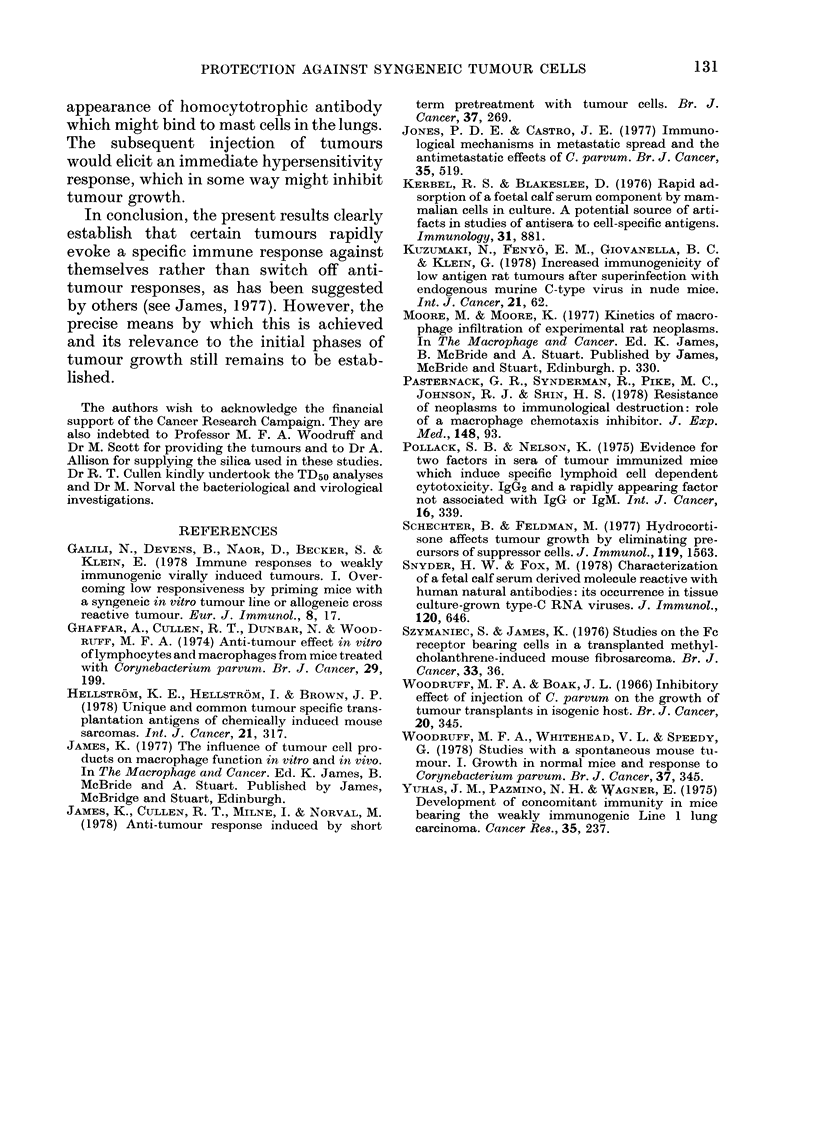# Further studies on antitumour responses induced by short-term pretreatment with syngeneic tumour cells.

**DOI:** 10.1038/bjc.1979.22

**Published:** 1979-02

**Authors:** K. James, I. Milne, J. Merriman, W. H. McBride

## Abstract

The ability of s.c. injected tumour cells to specifically inhibit the growth of similar cells injected i.v. 2 days later has been confirmed. The capacity of tumour cells to elicit this effect varies form tumour to tumour. Furthermore, it is more readily achieved with cultured than with freshly excised tumour cells. The superior effect elicited by cultured tumour cells was not overcome by treating them with trypsin or pronase. The protection achieved was impaired in T-cell-depleted mice and mice which had been irradiated (400 rad) prior to pretreatment. In contrast, it was not affected by administration of silica, sodium aurothiomolate or cortisone acetate. The results imply that T-cell-dependent responses are involved in the protection conferred by pre-injecting tumour cells shortly before i.v. challenge.


Br. J. Cancer (1979) 39, 122

### FURTHER STUDIES ON ANTITUMOUR RESPONSES INDUCED BY SHORT-TERM PRETREATMENT WITH SYNGENEIC TUMOUR CELLS

K. JAMES, I. MILNE, J. MERRIMAN AND W. H. McBRIDE*

*From the Departments of Surgery and *Bacteriology, University of Edinburgh Medical School, Teviot Place, Edinburgh EH8 9AG*

Received 2 October 1978 Accepted 30 October 1978

**Summary.—The ability of s.c. injected tumour cells to specifically inhibit the growth of similar cells injected i.v. 2 days later has been confirmed. The capacity of tumour cells to elicit this effect varies from tumour to tumour. Furthermore, it is more readily achieved with cultured than with freshly excised tumour cells. The superior effect elicited by cultured tumour cells was not overcome by treating them with trypsin or pronase. The protection achieved was impaired in T-cell-depleted mice and mice which had been irradiated (400 rad) prior to pretreatment. In contrast, it was not affected by administration of silica, sodium aurothiomolate or cortisone acetate. The results imply that T-cell-dependent responses are involved in the protection conferred by pre-injecting tumour cells shortly before i.v. challenge.**

RECENTLY, we reported that the pre-injection (s.c.) of syngeneic methylcholanthrene-induced fibrosarcoma cells inhibited the growth of homologous tumour injected i.v. 1 or 2 days later. This effect, however, was not observed if the s.c. injection was delayed until one day after tumour challenge. In addition, this pretreatment frequently enhanced the cytostatic effect of peritoneal-exudate cells on tumour-cell monolayers (James *et al.*, 1978). These observations were somewhat unexpected, in view of the many reports then appearing, suggesting that transplanted tumours might promptly release products which could impair the host response against themselves, so permitting the tumour to escape surveillance (reviewed in James, 1977).

In view of the effectiveness of the protection observed and the rapidity with which it was achieved, we decided this phenomenon was worth further investigation. The present report summarizes the results of studies undertaken; (a) to establish more conclusively the specificity of the effect, (b) to compare the protection conferred by tumour-cell inocula prepared in various ways, and (c) to ascertain the possible immunological mechanisms involved.

### MATERIALS AND METHODS

*Mice.*—All the investigations were performed in inbred CBA/Ca male mice age 10–12 weeks. These mice were bred from stock supplied by the MRC Laboratory Animals Centre, Carshalton, Surrey.

T-cell-deprived mice were prepared by thymectomizing 5-week-old mice and subjecting them 1 week later to 800 rad whole-body irradiation with thorax shielding. About 7 weeks later the animals were used. The immune status of the T-cell-deprived mice was routinely assessed by challenging them i.p. with $3 \times 10^8$ sheep erythrocytes and determining 7–9 days later the levels of circulating antibodies (both sensitive and resistant to mercaptoethanol) by standard passive haemagglutination techniques. The mice were also checked at the time of sacrifice for thymic remnants.

In certain instances, the mice were also subject to 400 rad whole-body X-irradiation with thorax shielding. A Westinghouse X-ray machine with the following physical conditions was used throughout for X-irradiation: 230 kV, 15 mA, with 0·5 mm Cu and 1·00 mm Al filters, a target-to-object distance of 75 cm and a delivery rate of 60 rad/min.

TABLE I.—*Some characteristics of the syngeneic tumours used in the present study*

| Tumour designation | Description | Generation Nos.* | How induced | Immunogenicity† | $TD_{50}$‡ |
|---|---|---|---|---|---|
| CCH1 | Fibrosarcoma | 21 or 22 | 3 methylcholanthrene | $10^5$–$10^6$ | 27 |
| CCH5 | Fibrosarcoma | 3 | 3 methylcholanthrene | $10^5$ | 830 |
| T3 | Fibrosarcoma | — | By transplantation of embryo cells spontaneously transformed *in vitro* | $10^4$ | 57 |
| W54 | Adenocarcinoma | 5 | Spontaneous | $<10^4$ | ND |

* Prior to transplantation or establishment in tissue culture. T3 was maintained in culture throughout.
† The challenge dose against which the preinjection of $10^6$ irradiated tumour cells 2 weeks earlier affects complete protection.
‡ The number of tumour cells which when injected s.c. gives rise to tumours in 50% of the mice.

*Tumours.*—The syngeneic tumours used included two MC-induced fibrosarcomas (designated CCH1 and CCH5), a spontaneous adenocarcinoma (W54) and a fibrosarcoma (T3) which had been obtained after injection of CBA mice with syngeneic embryo cells which had undergone spontaneous transformation *in vitro*. Further details on these tumours are in Table I and elsewhere (James *et al.*, 1978; Woodruff *et al.*, 1978). Cultured tumour cells were generally used for pretreatment and challenge. These cells had been maintained in culture for varying periods of time, under conditions previously described by Ghaffar *et al.*, (1974). The culture line was routinely examined for viral contamination, as previously described (James *et al.*, 1978) and shown to be free of viruses and other micro-organisms.

The tumour cells were generally harvested from the culture flasks by incubating the washed tumour-cell monolayers in Dulbecco A containing 0·2% (w/v) EDTA for 10 min. On certain occasions (see later) the cells were harvested by incubating for 3–5 min in Dulbecco/EDTA containing 0·5 mg/ml trypsin (Koch–Light, Colnebrook, England) whilst on other occasions cells recovered without the aid of trypsin were subjected to further treatment with pronase for 20 min at 37°C. The conditions used for treatment of the cultured tumour cells were similar to those used in preparing tumour-cell suspensions from freshly excised tumours, apart from the omission of treatment with deoxyribonuclease (see Woodruff & Boak, 1966).

*Other materials.*—The silica (Dorentrup Quartz Nr 12 1–5 $\mu$m) was sterilized by dry heating at 160°C for 2 h. It was then suspended in sterile 0·15M saline and vigorously mixed before injection. The cortisone acetate (Glaxo Pharmaceuticals Ltd, Greenford, England) was suspended at a concentration of 12·5 mg/ml in sterile 0·15M saline. Finally the sodium aurothiomalate (Myochrysine, 45% metallic gold; May and Baker Ltd, Dagenham) was diluted with sterile 0·15M saline to a concentration of 10 mg/ml.

In vivo *experimental model.*—The basic protocol involved injecting mice s.c. with $10^6$ syngeneic tumour cells 2 days before i.v. challenge with homologous tumour or other syngeneic tumours. The mice were killed 14 days after challenge, the lungs removed and fixed in Bouin's solution and the number of tumour nodules per lung counted. Each experimental group initially contained a minimum of 8 mice. Further experimental details are recorded elsewhere in the text or in the footnotes to tables and figures.

*Presentation of results.*—The number of tumour nodules observed in the lungs of individual mice has been presented in scattergram or tabular form and the significance of the results has been determined by the Wilcoxon Rank-Sum test. Values of $P<0·05$ were regarded as significant.

### RESULTS

### *Specificity of short-term protection*

Our initial studies suggested that significant inhibition of the growth of i.v. administered CCH1 tumour cells could only be achieved by the pre-injection of homologous tumour cells, other syngeneic and allogeneic tumours exerting little, if any, effect. The present experiments were performed to see whether specific protection could also be achieved in other tumour models. The results of these studies are presented in Figs. 1 and 2.

FIG. 1.—The effect on the growth of i.v. injected MC fibrosarcoma CCH5 of the pre-injection of various syngeneic tumour cells. Mice injected s.c. with $10^6$ cultured tumour cells on Day −2 and challenged i.v. on Day 0 with $5 \times 10^4$ cultured CCH5 tumour cells. 14 days after challenge the only significant reduction ($P < 0{\cdot}01$) was seen in mice pretreated with homologous tumour. Pretreatment: A—No pretreatment; B—CCH1; C—CCH5; D—T3; D—W54.

FIG. 2.—The effect on the growth of i.v. injected T3 of the preinjection of various syngeneic tumour cells. Mice injected s.c. with $10^6$ cultured tumour cells on Day −2 and challenged on Day 0 with $5 \times 10^4$ cultured T3 cells. None of the pretreatments significantly inhibited lung tumour nodule formation. Pretreatment as in legend for Fig. 1.

It will be observed from Fig. 1 that the lung-colonizing ability of i.v. injected CCH5 tumour cells was severely impaired by the s.c. injection of $10^6$ CCH5 cells 2 days prior to i.v. challenge. The pre-injection of other syngeneic tumour cells was without effect, thus confirming and extending our previous specificity studies with CCH1 tumour (James *et al.*, 1978).

However, additional studies revealed that the effect observed is not universal, for certain tumours (at least at the doses used) were not able to elicit a protective effect in this short-term pre-treatment model. This is illustrated in Fig. 2, where it can be seen that the s.c. injection of $10^6$ T3 tumour cells failed to inhibit the growth of T3 tumour injected i.v. 2 days later.

*A comparison of the protection achieved by tumour-cell suspensions processed in various ways*

An overall analysis of the data obtained in our original studies indicated that while a highly reproducible protection could be achieved after short-term pre-treatment with cultured tumour cells, a somewhat inconsistent effect was seen after the pre-injection of tumour-cell suspensions obtained by pronase digestion of freshly excised tumour cells (James *et al.*, 1978). We decided, therefore, to compare directly the ability of both cultured and freshly excised tumour cells to inhibit tumour nodule formation after challenge with

FIG. 3.—The effects on the growth of i.v. injected CCH1 tumour cells of the preinjection of cultured or freshly excised homologous tumour. Mice injected s.c. with $10^6$ cultured or freshly excised tumour cells on Day $-2$ and challenged i.v. on Day 0 with $5\times10^4$ cultured or freshly excised tumour cells. Preinjection of cultured cells significantly inhibited ($P<0{\cdot}01$) tumour nodule formation as measured 14 days after i.v. challenge with either tumour cell preparation, while the freshly excised preparation had no effect. Observe also the superior lung-colonizing properties of the freshly excised cells. Pretreatment: A and C—No pretreatment; B and D—Cultured cells; C and F—Freshly excised cells.

FIG. 4.—A comparison of the protective effects achieved by the preinjection of enzyme-treated and untreated cultured tumour cells and foetal calf serum. Mice were injected s.c. on Day $-2$ with $10^6$ enzyme-treated or untreated cultured CCH1 tumour cells or 0·1 ml of 10% (w/v) foetal calf serum, and challenged i.v. with $5\times10^4$ CCH1 tumour cells on Day 0. Both untreated and enzyme-treated cultured tumour cells significantly impaired tumour nodule formation, whereas the preinjection of foetal calf serum was without effect. Pretreatment: A—RPMI; B—RPMI plus FCS; C—Untreated tumour cells; D—Trypsin-treated tumour cells; E—Pronase-treated tumour cells.

either cultured or freshly excised tumour cells.

It will be seen from Fig. 3 that the lung-colonizing ability of freshly excised tumour cells was superior to that of cultured tumour cells. In addition, the tumours resulting from s.c. pre-treatment with freshly excised cells were larger (mean diam. 15·2 mm) than those after s.c. transplantation of cultured tumour cells (mean diam. 12·8 mm). Nevertheless, it is also readily apparent that cultured cells alone were capable of significantly inhibiting ($P<0{\cdot}01$) tumour-nodule formation after i.v. challenge with either freshly excised or cultured tumour cells.

Additional experiments were undertaken to ascertain whether the superior protective effect obtained with cultured cells might be reduced by submitting these cells to a similar enzyme treatment to that used in the preparation of tumour-cell suspensions from freshly excised tumours, a procedure which undoubtedly removes certain cell-surface antigens. These studies revealed that both pronase- and trypsin-treated cultured cells were as effective as non-enzyme-treated cultured tumour cells in eliciting the short-term protective effect (Fig. 4). Further studies undertaken in parallel with the above, indicated that the pre-injection of 0·1 ml of a 10% v/v solution of FCS in RPMI medium had no effect on the growth of tumour cells injected i.v. 2 days later.

FIG. 5.—The effect of silica treatment on the protection achieved by preinjecting tumour cells 2 days before i.v. challenge. Mice were injected daily on Days −4 to 0 with either 0·2 ml of physiological saline (Groups A and B) or 0·2 ml of saline containing 2·5 mg silica (Groups C and D). These injections were given by both the i.p. (Days −4, −2 and 0) and the i.v. routes (−3 and −1). Mice in Groups B and D were also injected s.c. with $10^6$ cultured CCH1 fibrosarcoma cells on Day −2. All mice were challenged i.v. on Day 0 with $5 \times 10^4$ cultured CCH1 cells, and the number of tumour nodules per lung counted 14 days later. The protection conferred by preinjection of tumour cells was not overcome by silica treatment. Pretreatment; A—Saline; B—Saline plus tumour; C—Silica alone; D—Silica plus tumour.

*Possible mechanisms whereby short-term protection is achieved*

A number of experiments have been undertaken to help clarify the possible means whereby protection is conferred in this short-term pre-treatment model. Although these experiments failed to establish the precise mechanism involved, they nevertheless have enabled us to exclude some of the possible explanations originally advanced (James *et al.*, 1978).

In order to assess the possible role of macrophages we compared the protection achieved in normal mice and mice receiving a course of silica injections, which is known to severely impair macrophage function (*e.g.* Jones and Castro, 1977). In essence this involved injecting mice i.p. with 2·5 mg of silica in 0·2 ml of physiological saline, on Days −4, −2 and 0, relative to i.v. challenge with tumour cells. Additional i.v. injections of 2·5 mg of silica were also given on days −3 and −1. As indicated in Fig. 5, this intensive course of silica treatment failed to influence the protection due to short-term pre-treatment with homologous tumour cells. Further studies revealed that the protection was also not ablated by treatment with sodium auriothiomalate (1 mg/day on Days −5 to 0) adding further support to the contention that the observed effect was not macrophage mediated (see Fig. 6).

The possible involvement of T-dependent immune processes in this phenomenon was investigated in B-cell-deprived mice. In these experiments, mice which had been T-cell-depleted as described earlier were injected s.c. after a 7–8-week interval with $10^6$ lethally irradiated (22,000 rad) or untreated cultured CCH1 tumour cells and challenged i.v. with $10^6$ cultured CCH1 tumour cells 2 or 11 days

FIG. 6.—The effect of sodium aurothiomalate treatment on the protection achieved by preinjecting tumour cells 2 days before i.v. challenge. Mice in Groups C and D received daily i.p. injection of 1 mg of sodium aurothiomalate on Days −5 to 0. In addition, animals in Groups B and D were injected s.c. with $10^6$ cultured CCH1 fibrosarcoma cells on Day −2. All mice were challenged i.v. with $5 \times 10^4$ cultured CCH1 fibrosarcoma cells on Day 0 and the number of tumour nodules per lung was counted 14 days later. The protective effect conferred by pre-injection of tumour cells on Day −2 was not overcome by sodium aurothiomalate treatment. Pretreatment: A—No pretreatment; B—Tumour alone; C—Sodium aurothiomalate alone; D—Sodium aurothiomalate and tumour.

later. Similar experiments were simultaneously undertaken in intact or sham-thymectomized X-irradiated mice. The number of lung tumour nodules seen 2 weeks after challenge is indicated in Table II. It will be seen that in T-cell-deprived mice the s.c. injection of tumour cells fails to inhibit significantly the growth of tumour cells injected i.v. 2 days later. This contrasts markedly with the effect noted in intact or sham-thymectomized X-irradiated mice. It should also be stressed that serological analyses revealed that the capacity of the T-depleted mice to mount a 7S antibody (mercaptoethanol resistant) response to sheep erythrocytes was severely impaired relative to that seen in sham-thymectomized X-irradiated mice and intact mice. This confirms the effectiveness of the T-cell-depletion procedure.

Whilst the above observations revealed that T cells were involved in short-term protection, additional studies in cortisone-treated mice showed that the cortisone-sensitive T-cell subset did not play a crucial role (Table III). However, protection was somewhat impaired (though not ablated) in mice which had been subjected to 400 rad whole-body irradiation before s.c. pre-treatment (Table IV).

It should be noted that throughout these studies the pre-injection of $10^6$ viable syngeneic tumour cells almost always produced tumours with diameters of 10 mm or more when they were killed 14 days after i.v. challenge.

### DISCUSSION

The present results confirm our initial observations that the pre-injection of syngeneic tumour cells into mice shortly before i.v. challenge with the same tumour impairs the development of lung tumour nodules. They also indicate that (a) the effect is specific and more readily achieved with some tumours than others, (b) cultured tumour cells are more effective than freshly excised cells at evoking protection and (c) T-dependent cells, though not macrophages, play a crucial role in this phenomenon.

Why this short-term protection can be readily achieved with the 2 MC-induced fibrosarcomas and not with the T3 fibrosarcoma remains to be established. It may simply reflect a difference in the immunizing capacity of the tumours, for the 2 MC fibrosarcomas are known to be more im-

TABLE II.—*A comparison of the effects of pretreatment at various times on lung tumour metastasis in B mice*

| Exp. | Group | Immune status | Day* tumour cells injected s.c. | Tumour nodules/lung | P† |
|---|---|---|---|---|---|
| 1 | A | | Not injected | 3, 5, 6, 7, 7, 9, 10, 11, 12 | |
| | B | Intact | −2 | 0, 0, 0, 1, 1, 2, 4, 4, | <0·01 |
| | C | | −11 | 0, 0, 0, 0, 0, 0, 0, 0, 2 | <0·01 |
| | D | Sham | Not injected | 1, 7, 7, 8, 8, 9, 15, 16, 18, 28 | |
| | E | thymectomized | −2 | 2, 3, 4, 4, 4, 5, 5, 7, 7, 10 | 0·02 |
| | F | and irradiated | −11 | 0, 0, 0, 0, 0, 0, 0, 1, 1, 1 | <0·01 |
| | G | Thymectomized | Not injected | 1, 8, 9, 11, 12, 15, 15, 16, 16, 18 | |
| | H | and irradiated | −2 | 1, 1, 3, 3, 3, 3, 7, 9, 14, 19 | >0·05 |
| | I | | −11 | 0, 0, 0, 0, 1, 2, 3, 3, 5, 8, 11 | <0·01 |
| 2 | A | | Not injected | 133, 211, 232, 241, 242, 260, 270, 347, 350, 455 | |
| | B | Intact | −2 | 0, 0, 0, 0, 0, 0, 1, 1, 198 | <0·01 |
| | C | | −11 | 0, 0, 0, 0, 0, 0, 0, 1, 1 | <0·01 |
| | D | Sham | Not injected | 12, 13, 102, 169, 173, 183, 201, 222, 300 | |
| | E | thymectomized | −2 | 0, 3, 14, 25, 45, 58, 101, 108, 134, 157 | 0·05 |
| | F | and irradiated | −11 | 0, 0, 1, 16, 16, 21, 72, 74, 79, 114 | 0·05 |
| | G | Thymectomized | Not injected | 142, 143, 254, 270, 306, 400, 438 | |
| | H | and irradiated | −2 | 12, 13, 102, 169, 173, 183, 201, 222, 300 | >0·05 |
| | I | | −11 | 6, 9, 14, 150, 164, 178, 300, 304, 330, 340 | 0·10 |

* The mice were injected s.c. with $10^6$ cultured irradiated (Exp. 1) or unirradiated (Exp. 2) CCHl tumour cells either 11 days or 2 days prior to i.v. challenge with $1 \times 10^5$ (Exp. 1) or $2 \times 10^5$ (Exp. 2) cultured CCHl tumour cells. The i.v. challenge in all groups were given on the same day.

† All values compared with respective non-pretreated counterparts.

Note that the protection achieved in the pretreated thymectomized group is less marked than that in the pretreated intact and sham-thymectomized groups.

TABLE III.—*Effect of cortisone acetate (CA) on the protection conferred by short-term preinjection of tumour cells**

| Exp. | Group | Treatment | Tumour nodules/ lung | P |
|---|---|---|---|---|
| 1 | A | CA alone | 13, 29, 32, 41, 45, 88, 96, 217, 243, 297 | |
| | B | CA+ tumour cells | 0, 0, 0, 17, 23, 24, 28, 30, 35, 88 | <0·01 |
| 2 | A | CA alone | 17, 25, 40, 42, 52 76 | |
| | B | CA+ tumour cells | 0, 0, 0, 0, 0, 10, 15, 26 | <0·01 |
| 3 | A | CA alone | 85, 219, 232, 254, 276, 304, 354, 398, 444, 493 | <0·01 |
| | B | CA+ tumour cells | 14, 19, 22, 44, 83, 94, 126, 345 | |

* In both groups 2·5 mg of cortisone acetate in 0·2 ml of saline was administered i.p. on Days −3, −1 and +1. Group B was also injected s.c. with $10^6$ CCHl fibrosarcoma cells on Day −2. All mice were challenged i.v. with $10^5$ CCHl cells on Day 0, and the number of tumour nodules counted on Day 14.

In all experiments the preinjection of homologous tumour cells significantly inhibited the number of tumour nodules produced by the i.v. injection of tumour cells munogenic than the T3 tumour (see Table I). Furthermore, as the protection elicited by CCHl is dose dependent (see James *et al.*, 1987) it is possible that higher doses of T3 might have proved effective.

The reason why cultured CCHl fibrosarcoma cells evoke a better protection than freshly excised cells is still far from clear. However, it should be noted that differences in host response to syngeneic cultured and freshly excised tumour cells have been noted by others. For example, recent reports indicate that cultured tumour cells elicit a more rapid infiltration of host cells than do their freshly excised counterparts (Moore and Moore, 1977) are more susceptible to immunological destruction *in vivo* (Pasternack *et al.*, 1978) and may also generate a more significant cytotoxic response (Galili *et al.*, 1978). In addition, in our own laboratory we have seen differences in the growth rates of freshly excised and cultured tumours (see Fig. 3) and in their host immunoglobulin content (Bessos, Merriman and James, unpublished observations).

TABLE IV.—*Effect of prior irradiation (400 rad) on the protection conferred by short-term preinjection of tumour cells*

| | | Treatment | | | |
|---|---|---|---|---|---|
| Exp. | Group | Tumour cells s.c.* | Day irradiated† | Tumour nodules/lung | P |
| 1 | A | No | Not irradiated | 37, 43, 85, 131, 135, 140, 142, 160, 163 | 0·02 |
| | B | Yes | | 0, 0, 2, 5, 5, 14, 33, 40, 88, 300+ | |
| | C | No | −3 | 63, 240, 300+, 300+, 300+ | |
| | D | Yes | −3 | 15, 26, 110, 138, 183, 195, 202 | ≃0·05 |
| | E | No | −1 | 72, 124, 190, 200, 200, 250, 270, 280, 300,+ 300+ | <0·01 |
| | F | Yes | −1 | 5, 17, 32, 57, 69, 120, 130, 131, 137, 170 | |
| | G | No | +1 | 120, 180, 220, 260, 270, 270, 300+, 300+ | <0·01 |
| | H | Yes | +1 | 0, 5, 23, 26, 41, 73, 73, 87, 113, 208 | |
| 2 | A | No | Not irradiated | 34, 46, 47, 77, 128, 146, 172, 190, 300+ | <0·01 |
| | B | Yes | | 0, 0, 0, 0, 0, 1, 2, 12, 13 | |
| | C | No | −3 | 116, 117, 131, 176, 180, 194, 220, 252 | ≃0·05 |
| | D | Yes | −3 | 0, 12, 50, 94, 120, 123, 234, 300+ | |
| | E | No | −1 | 146, 197, 210, 245, 246, 268, 279, 309 | 0·02 |
| | F | Yes | −1 | 0, 0, 0, 0, 10, 15, 33, 59, 144 | |
| | G | No | +1 | 86, 103, 146, 158, 180, 193, 205, 206, 278, 278 | <0·01 |
| | H | Yes | +1 | 0, 0, 2, 2, 5, 18, 44, 78, 118 | |

* $10^6$ CCHl tumour cells injected s.c. 2 days before i.v. challenge on Day 0 with $10^5$ CCHl tumour cells.
† Day 400 rad whole-body X-irradiation (thorax shielded) performed in relationship to i.v. challenge on Day 0.
Though the protective effect was not ablated by irradiation, it was less pronounced in animals irradiated before s.c. injection of tumour cells.

Initially, we were inclined to the view that the observed effects were due to inherent differences in the antigenicity of the 2 preparations. One possibility was that the use of proteolytic enzymes in the preparation of tumour-cell suspensions had stripped tumour-specific transplantation antigens from the cell surface. Although such antigens would eventually be regenerated after s.c. transplantation, the delay in their expression would most likely produce a delay in the stimulation of host defence mechanisms. Thus, by the time they were eventually expressed, the i.v. injected tumour would be irreversibly established. An alternative explanation is that the cultured tumour cells had absorbed foetal-calf serum proteins from the culture media and these exogenous proteins modulated the immune response. In this context it is interesting to note that others have reported that mammalian cells (Kerbel and Blakeslee, 1976) and virus (Snyder and Fox, 1978) may absorb foetal calf serum proteins from culture media. Such absorbed proteins may evoke a cellular or humoral response against themselves.

The observation that the trypsin or pronase treatment of cultured cells fails to modify the protective effect they elicit suggests, at least indirectly, that the inferior effect of freshly excised cells is probably not due to the removal of antigens by these enzymes during cell preparation. Nevertheless, it must be borne in mind that deoxyribonuclease treatment was also used in the preparation of cells from freshly excised tumours and this could conceivably have altered the properties of the cells.

The alternative explanation, that the effects may be due to foetal-calf serum proteins absorbed during prolonged culture, is also unlikely. In the first place, the protective effect is tumour specific and in the second, the effect cannot be reproduced by injecting a relatively large dose of foetal calf serum 2 days before i.v. challenge. It is conceivable, however, that certain foetal-calf serum proteins are integrated into the tumour-cell surface, and that their presence augments the immunogenicity of tumour-specific transplantation antigens, in the same way that viral antigens are believed to potentiate the immune response to weakly immunogenic tumours (Hellstrom *et al.*, 1978; Kuzamaki *et al.*, 1978).

While the above suggestions are of theoretical interest we feel that the differences are most probably due to the presence in the freshly excised tumour-cell preparations of host lymphoreticular cells, which somehow modulate the immune response to the tumour cells after transplantation into a new host. For example, in the light of previous observations from our laboratory on the CCH1 tumour used in these studies (Szymaniec and James, 1976) it is conceivable that freshly excised tumour-cell suspension contained suppressor cells (either T cells or macrophages) which interfered with the development of prompt and effective anti-tumour immunity. In this context it could be argued that the superior lung-colonizing properties of freshly excised tumour, over their cultured counterparts (see Fig. 3), was due to the presence of suppressor host cells in the freshly excised tumour-cell suspension. It should be noted however that others have recently shown that differences in the growth characteristics of freshly excised and cultured tumour cells are related to their capacity to produce inhibitors of macrophage chemotaxis (Pasternack *et al.*, 1978).

An additional possibility is that the larger tumours which arise after transplantation of freshly excised cells give less protection due to an "eclipse" phenomenon. Whatever the explanation for the differences in host responses to freshly excised and cultured tumour cells, these recent observations are undoubtedly of importance in relation to all studies using tumour isotransplants, and certainly warrant further investigation.

Although the mechanisms by which short-term protection is achieved still remains to be established, our observations to date indicate that T-cell-dependent immune responses are important, and that macrophages probably play an insignificant role. Furthermore, the fact that the effect can be achieved in cortisone acetate-treated mice suggests that a steroid-resistant T-cell population is involved, thus excluding a role for steroid-sensitive suppressor-cell precursors (Schechter and Feldman, 1977). Whatever the cells involved, our studies indicate that a radiation-sensitive precursor exists. The mature effector cells, however, are resistant to low doses of X-irradiation. It should be stressed that the protective effect conferred may be yet another example of concomitant immunity. Nevertheless, it is interesting to note that the protection conferred is not related to the size of the s.c. tumour at the time of sacrifice, and can be readily achieved in the absence of a tumour burden, namely after s.c. injection of lethally irradiated tumour cells (*e.g.* Table II and James *et al.*, 1978). In addition it should be noted that while the s.c. injection of tumour as late as one day before i.v. challenge inhibits the growth of lung tumour nodules, such treatment is ineffective if delayed until the time of i.v. challenge, even though there is no detectable difference in the size of the s.c. tumours when the animals are killed (see James *et al.*, 1978). Finally, whilst we have not performed studies to see whether the pre-injection of tumour cells i.v. can inhibit the growth of tumour cells injected s.c., such effects have been reported by others (Yuhas *et al.*, 1975).

Our studies to date do not exclude the involvement of antibody-mediated processes. Among the untested possibilities are the rapid development of antibodies which might render the i.v. injected tumours susceptible to either complement-mediate lysis or antibody-dependent cellular cytotoxicity mechanisms. In this connection it is interesting to note that others have observed the rapid appearance, within 2 days of tumour injection, of humoral factors which are capable of inducing antibody-dependent cellular cytotoxic reactions (Pollack and Nelson, 1975). Finally, the possibility also exists that pre-injection of tumour results in the rapid appearance of homocytotrophic antibody which might bind to mast cells in the lungs. The subsequent injection of tumours would elicit an immediate hypersensitivity response, which in some way might inhibit tumour growth.

In conclusion, the present results clearly establish that certain tumours rapidly evoke a specific immune response against themselves rather than switch off anti-tumour responses, as has been suggested by others (see James, 1977). However, the precise means by which this is achieved and its relevance to the initial phases of tumour growth still remains to be established.

The authors wish to acknowledge the financial support of the Cancer Research Campaign. They are also indebted to Professor M. F. A. Woodruff and Dr M. Scott for providing the tumours and to Dr A. Allison for supplying the silica used in these studies. Dr R. T. Cullen kindly undertook the $TD_{50}$ analyses and Dr M. Norval the bacteriological and virological investigations.

### REFERENCES

GALILI, N., DEVENS, B., NAOR, D., BECKER, S. & KLEIN, E. (1978 Immune responses to weakly immunogenic virally induced tumours. I. Overcoming low responsiveness by priming mice with a syngeneic *in vitro* tumour line or allogeneic cross reactive tumour. *Eur. J. Immunol.*, **8**, 17.

GHAFFAR, A., CULLEN, R. T., DUNBAR, N. & WOODRUFF, M. F. A. (1974) Anti-tumour effect *in vitro* of lymphocytes and macrophages from mice treated with *Corynebacterium parvum*. *Br. J. Cancer*, **29**, 199.

HELLSTRÖM, K. E., HELLSTRÖM, I. & BROWN, J. P. (1978) Unique and common tumour specific transplantation antigens of chemically induced mouse sarcomas. *Int. J. Cancer*, **21**, 317.

JAMES, K. (1977) The influence of tumour cell products on macrophage function *in vitro* and *in vivo*. In *The Macrophage and Cancer*. Ed. K. James, B. McBride and A. Stuart. Published by James, McBridge and Stuart, Edinburgh.

JAMES, K., CULLEN, R. T., MILNE, I. & NORVAL, M. (1978) Anti-tumour response induced by short term pretreatment with tumour cells. *Br. J. Cancer*, **37**, 269.

JONES, P. D. E. & CASTRO, J. E. (1977) Immunological mechanisms in metastatic spread and the antimetastatic effects of *C. parvum*. *Br. J. Cancer*, **35**, 519.

KERBEL, R. S. & BLAKESLEE, D. (1976) Rapid adsorption of a foetal calf serum component by mammalian cells in culture. A potential source of artifacts in studies of antisera to cell-specific antigens. *Immunology*, **31**, 881.

KUZUMAKI, N., FENYÖ, E. M., GIOVANELLA, B. C. & KLEIN, G. (1978) Increased immunogenicity of low antigen rat tumours after superinfection with endogenous murine C-type virus in nude mice. *Int. J. Cancer*, **21**, 62.

MOORE, M. & MOORE, K. (1977) Kinetics of macrophage infiltration of experimental rat neoplasms. In *The Macrophage and Cancer*. Ed. K. James, B. McBride and A. Stuart. Published by James, McBride and Stuart, Edinburgh. p. 330.

PASTERNACK, G. R., SYNDERMAN, R., PIKE, M. C., JOHNSON, R. J. & SHIN, H. S. (1978) Resistance of neoplasms to immunological destruction: role of a macrophage chemotaxis inhibitor. *J. Exp. Med.*, **148**, 93.

POLLACK, S. B. & NELSON, K. (1975) Evidence for two factors in sera of tumour immunized mice which induce specific lymphoid cell dependent cytotoxicity. $IgG_2$ and a rapidly appearing factor not associated with IgG or IgM. *Int. J. Cancer*, **16**, 339.

SCHECHTER, B. & FELDMAN, M. (1977) Hydrocortisone affects tumour growth by eliminating precursors of suppressor cells. *J. Immunol.*, **119**, 1563.

SNYDER, H. W. & FOX, M. (1978) Characterization of a fetal calf serum derived molecule reactive with human natural antibodies: its occurrence in tissue culture-grown type-C RNA viruses. *J. Immunol.*, **120**, 646.

SZYMANIEC, S. & JAMES, K. (1976) Studies on the Fc receptor bearing cells in a transplanted methylcholanthrene-induced mouse fibrosarcoma. *Br. J. Cancer*, **33**, 36.

WOODRUFF, M. F. A. & BOAK, J. L. (1966) Inhibitory effect of injection of *C. parvum* on the growth of tumour transplants in isogenic host. *Br. J. Cancer*, **20**, 345.

WOODRUFF, M. F. A., WHITEHEAD, V. L. & SPEEDY, G. (1978) Studies with a spontaneous mouse tumour. I. Growth in normal mice and response to *Corynebacterium parvum*. *Br. J. Cancer*, **37**, 345.

YUHAS, J. M., PAZMINO, N. H. & WAGNER, E. (1975) Development of concomitant immunity in mice bearing the weakly immunogenic Line 1 lung carcinoma. *Cancer Res.*, **35**, 237.